# Docking Studies and α-Substitution Effects on the Anti-Inflammatory Activity of β-Hydroxy-β-arylpropanoic Acids

**DOI:** 10.3390/molecules16086645

**Published:** 2011-08-05

**Authors:** Jelena S. Savić, Sanda P. Dilber, Bojan D. Marković, Marina T. Milenković, Sote M. Vladimirov, Ivan O. Juranić

**Affiliations:** 1 Department of Pharmaceutical Chemistry, Faculty of Pharmacy, University of Belgrade, Vojvode Stepe 450, 11221 Belgrade, Serbia; 2 Department of Organic Chemistry, Faculty of Pharmacy, University of Belgrade, Vojvode Stepe 450, 11221 Belgrade, Serbia; 3 Department of Molecular Biology and Immunology, University of Belgrade, Vojvode Stepe 450, 11221 Belgrade, Serbia; 4 Department of Organic Chemistry, Faculty of Chemistry, University of Belgrade, Box 158, 11001, Belgrade, Serbia

**Keywords:** β-hydroxy-β-arylpropanoic acids, Reformatsky reaction, anti-inflammatory activity, molecular docking simulations, COX-2 selective inhibitor

## Abstract

Six β-hydroxy-β-aryl propanoic acids were synthesised using a modification of Reformatsky reaction which has already been reported. These acids belong to the aryl propanoic acid class of compounds, structurally similar to the NSAIDs, such as ibuprofen, and an anti-inflammatory activity is thus expected. The aim of this work was to determine anti-inflammatory activity, examine gastric tolerability, and to carry out molecular docking experiments to identify potential COX-2 inhibitors among the β-hydroxy-β-aryl propanoic acids, and to elucidate the effect α-methyl substitution on the anti-inflammatory activity. Anti-inflammatory activity and gastric tolerability were determined on rats using carragenan induced paw oedema method, and docking studies were carried out using Autodock v4.0.1. The range of ED_50_ values is between 127 µmol/kg and 15 µmol/kg, while the result for ibuprofen is 51.7 µmol/kg. Only slight hyperaemia or few petechiae were spotted on rat’s stomach. The results indicate that all compounds possess significant anti-inflammatory activity after oral administration, and that 2-methyl-3-hydroxy-3,3-diphenyl-propanoic acid has greatest activity, surpassing that of ibuprofen, a standard NSAID. Another compound, 3-hydroxy-3,3-diphenylpropanoic acid, shows activity matching that of ibuprofen, and is non-chiral and is proven to be non-toxic. The most of investigated compounds have interactions with P3 anchor site like COX-2 selective inhibitors. No tested substances or ibuprofen produced any significant gastric lesions.

## 1. Introduction

Cyclooxygenase (COX), also known as prostaglandin endoperoxide synthase, is a bifunctional enzyme that catalyzes the conversion of arachidonic acid to prostaglandin (PG) H_2_, the immediate precursor to prostaglandins, thromboxane and prostacyclin. The conversion of arachidonic acid into PGH_2_ proceeds through two separate reactions in which two molecules of O_2_ are incorporated into arachidonic acid bound in the COX site to form PGG_2_, which then diffuses to the peroxidase site (POX) to undergo a two-electron reduction to form the final product PGH_2_. Prostaglandins (PGs) are the lipid mediators made by most cells in the body except by red blood cells and released upon almost any type of chemical or mechanical stimulus [1]. The two definitely known isoforms of COX, named COX-1 and COX-2 show distinct expressions patterns and distinct biological activities. COX-1 Is formed in many different cells to create prostaglandins that serve for basic "housekeeping" messages throughout the body. This is a constitutively expressed protein that is responsible for the physiological production of prostaglandins. The COX-1 variant protein, named COX-3, is sensitive to inhibition with paracetamol [2]. COX-2 is only formed in special cells and is used for signalling both the pain and inflammation. This isoform is also called inducible isoform of enzyme COX [3]. In inflammatory processes COX-2 is overexpressed. 

Non-steroidal anti-inflammatory drugs (NSAIDs) are COX inhibitors and prevent PG synthesis, thus exhibiting analgesic, antipyretic and anti-inflammatory actions. However, NSAIDs have a number of adverse effects, mainly because of their inhibition of the constitutive isoform of COX. The major adverse effects of NSAIDs are gastrotoxic (e.g., damage of gastric mucosa, may provoke gastric bleeding and gastroduodenal ulcers), increased bleeding tendency and delay of the birth process [1]. Nowadays, there are known two types of COX inhibitors, nonselective, *i.e*., both COX-1 and COX-2 inhibitors, and predominantly selective COX-2 inhibitors such as the "coxibs". Since selective COX-2 inhibitors fail to inhibit constitutive COX-1 isoform, they have no gastrointestinal adverse effects. However, recent publications have suggested that COX-2 inhibitors, like rofecoxib and celecoxib, may be prothrombic and increase the risk of myocardial infarction [2]. Consequently, a synthesis of new NSAIDs, with potent anti-inflammatory, analgesic and antipyretic action, but with no adverse effects is highly desired. 

We have synthesized six β-hydroxy-β-aryl propanoic acids having none, one, or two methyl groups at the α-position. These acids are structurally similar to COX inhibitors: *p*-isobutylphenyl acetic acid (ibufenac), 4-biphenylacetic acid (felbinac), α-(4-isobutylphenyl)propanoic acid (ibuprofen), α-(6-methoxy-2-naphtyl)propanoic acid (naproxen). The compounds studied in this work are among compounds studied in our previous article and they affected the survival of HeLa cells (Human cervix epithelial adenocarcinoma cells), having IC_50_ values from 62.20 to 185.55 μM/L. Generally, the examined compounds did not affect proliferation of healthy human peripheral mononuclear cells (PBMC) IC50 > 300 μM/L [4]. Some of synthesized acids exhibit anti-inflammatory activity [5]. In this work, docking studies and α-substitution effect on the anti-inflammatory activity and gastric tolerability of the synthesized compounds were evaluated.

## 2. Results and Discussion

**Scheme 1:**
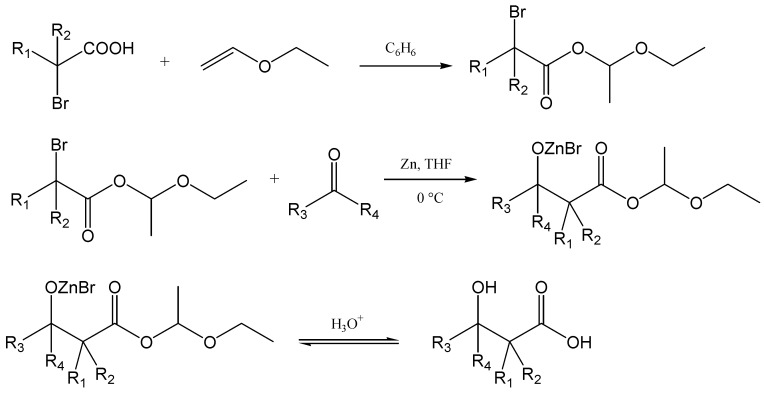
Synthetic sequence of β-hydroxy-β-arylpropanoic acids.

β-Hydroxy-β-aryl propanoic acids were synthesized by the two-step reaction. In the first step α-bromoalkanoic acid 1-ethoxyethyl ester intermediates were synthesized. The second step involved a Reformatsky reaction in tetrahydrofuran (THF) at –5 to 10 °C between the previously synthesized intermediates and 4-acetylbiphenyl or benzophenone in the presence of Zn (Scheme 1).

The syntheses and antiproliferative activity of some β-hydroxy-β-arylpropanoic acids were previously reported [4,5]. 3-Hydroxy-2-methyl-3-(4-diphenylyl)butanoic acid (**2**) was prepared by the general method described above and fully characterized. The resulting mixture of diastereomers was not separated. We observed a single spot on TLC, and assumed to have obtained a *threo* isomer, which has optimal geometry containing an intramolecular hydrogen bond and the most distant methyl groups.

**Figure 1:**
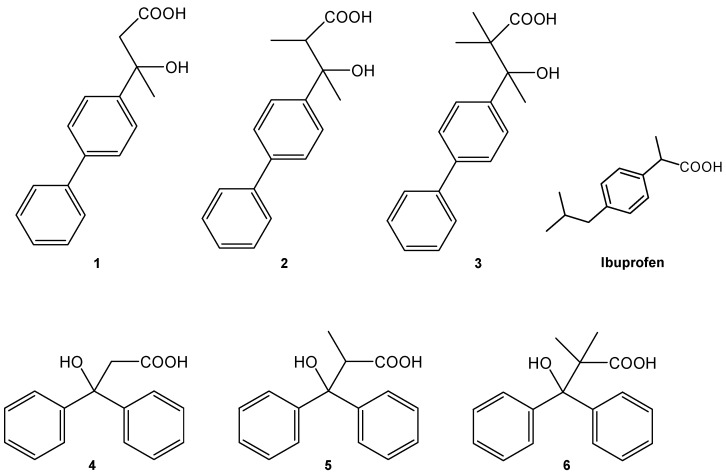
Structures of the studied compounds.

Synthesized acids shown in Figure 1 are 3-hydroxy-3-(4-diphenylyl)butanoic acid (**1**), 3-hydroxy-2-methyl-3-(4-diphenylyl)butanoic acid (**2**), 3-hydroxy-2,2-dimethyl-3-(4-diphenylyl)butanoic acid (**3**), 3-hydroxy-3,3-diphenylpropanoic acid (**4**), 3-hydroxy-3,3-diphenyl-2-methylpropanoic acid (**5**) and 3-hydroxy-3,3-diphenyl-2.2-dimethylpropanoic acid (**6**). 

### 2.1. Anti-inflammatory activity: carragenan-induced paw-oedema 

**Figure 2:**
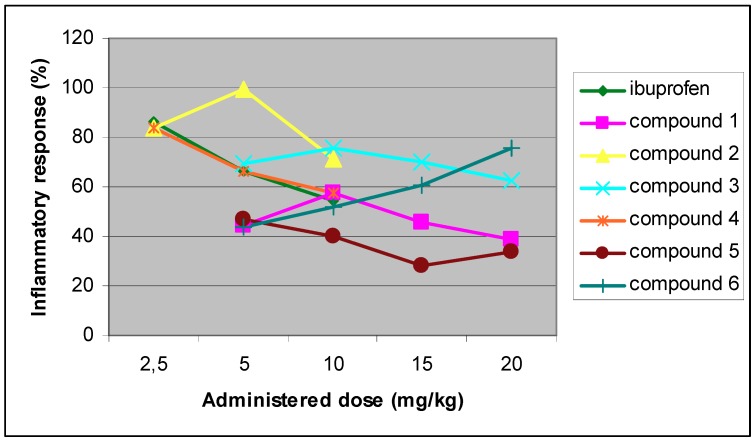
Dose dependence of anti-inflammatory effect of studied compounds listed on legend.

Results showed that all compounds (save compound **3**) produced significant, dose-dependent (like ibuprofen) anti-inflammatory effect (Figure 2). ED_50_ Values were calculated for ibuprofen and for our six compounds (Table 1). 

### 2.2. Gastric tolerability 

None of the tested substances (including ibuprofen) produced any significant gastric lesions. The changes observed were in range of 0–1 according to the Adami’s scoring scale. Namely, only slight hyperemia or few petechiae were registered in rat stomach regardless of given dose. 

### 2.3. Molecular docking computational experiments 

Table 1 shows the binding energies of the top scored solutions found during docking experiments of compounds **1–6** and ibuprofen (specific ligand for COX-1), along with ED_50_ values (μM/kg) obtained in the carrageenan-induced rat paw oedema test. In this table, the result for SC-558 (a specific ligand for COX-2) was not included, because of having a markedly different structure and the lack of a biological assay for it. 

**Figure 3:**
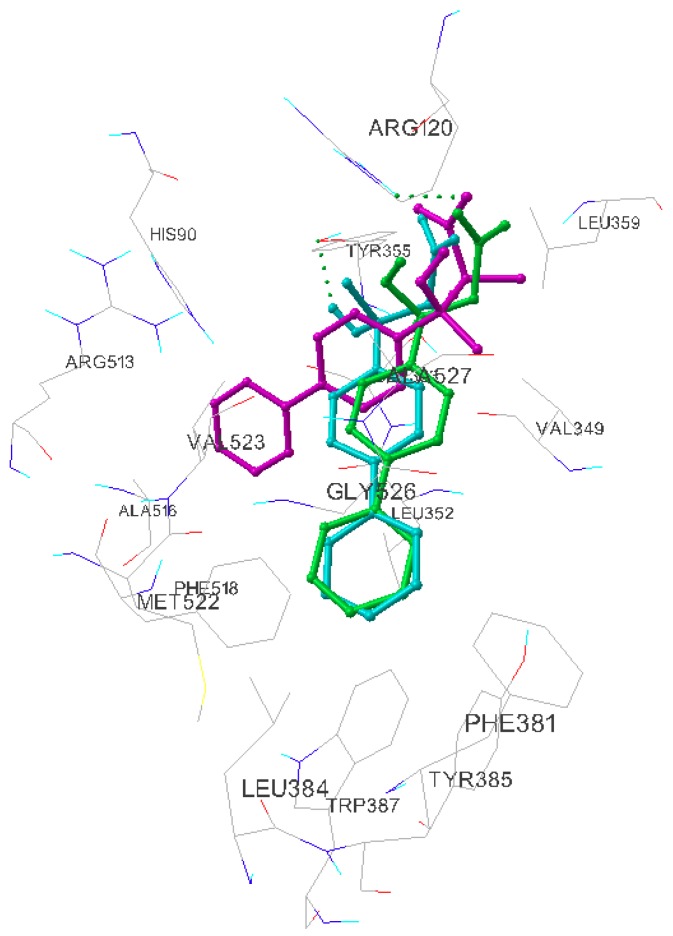
Superimposition of most favorable conformations of compounds **1** (green), **2** (blue) and **3** (magenta) docked into binding site of COX-2 receptor [6].

Analysis of docking results reveals the modes of the binding between ligands and active site of enzymes. Acids **1**, **2** and **3** form strong interactions, which includes salt bridges with Arg120 (**1**-1.67 Ǻ, **2**-1.92 Ǻ and **3**-1.95 Ǻ) and hydrogen bonds with phenolic hydroxyl of Tyr 355 (1-2.7 Ǻ, **2**-2.03 **Ǻ** and **3**-4.9 **Ǻ**), in first anchor site, P_1_. Compounds **1**, **2** and **3** form similar hydrophobic interactions with Ser 353 (~3 Ǻ) and with Val 349 (~3 Ǻ) but acid **1** forms π→ cation interactions with residues His 90, Arg 513 and Gln 192 (~3 Ǻ), (Figure 3). 

Acids **4**, **5** and **6** form interactions with amino acid residues into all three cyclooxygenase anchor sites and they act as selective COX-2 inhibitors.

Molecular docking experiments gave a reasonable explanations for different anti-inflammatory activity of synthesized β-hydroxy-β-arylpropanoic acids. All synthesized compounds show improved binding energies compared to the active ibuprofen. Differences in binding energies of enantiomers are not significant. The carboxylate terminal groups of these molecules differ primarily in sterical hindrance caused by the presence or absence of an α- and β-methyl group(s). Despite these differences, the carboxylate groups of all compounds are essentially superimposable. The inhibitor carboxylate participates in a network of polar interactions, which includes salt bridges between the inhibitor and Arg 120 and hydrogen bonds of the inhibitor and phenolic hydroxyl of Tyr 355 (first anchor site, P_1_). These amino acid residues together with His 90 and Glu 524 tightly lock inhibitors into cyclooxygenase active site [6]. Methyl groups form hydrophobic interactions with Ser 353 and Val 349 (<4 Ǻ). Substituents attached to a phenyl ring placed in β-position in respect to COOH of inhibitors (isobutyl group of ibuprofen, second phenyl ring of all compounds) lie in hydrophobic cleft (second anchor site, P_2_) that is lined with Leu 352, Tyr 385, Trp 387, Phe 518, Gly 526 and Ser 530 residues. 

Methyl groups, particularly two methyl groups at the α-position, move the whole molecule towards the hydrophilic pocket (the third anchor site, P_3_) in which they form interactions like COX-2 selective inhibitors [7]. At the same time, methyl groups send away the carboxylate groups from Arg 120 and Tyr 355 influence on reducing of hydrophobic interactions and decrease of activity of the synthesized acids.

**Figure 4:**
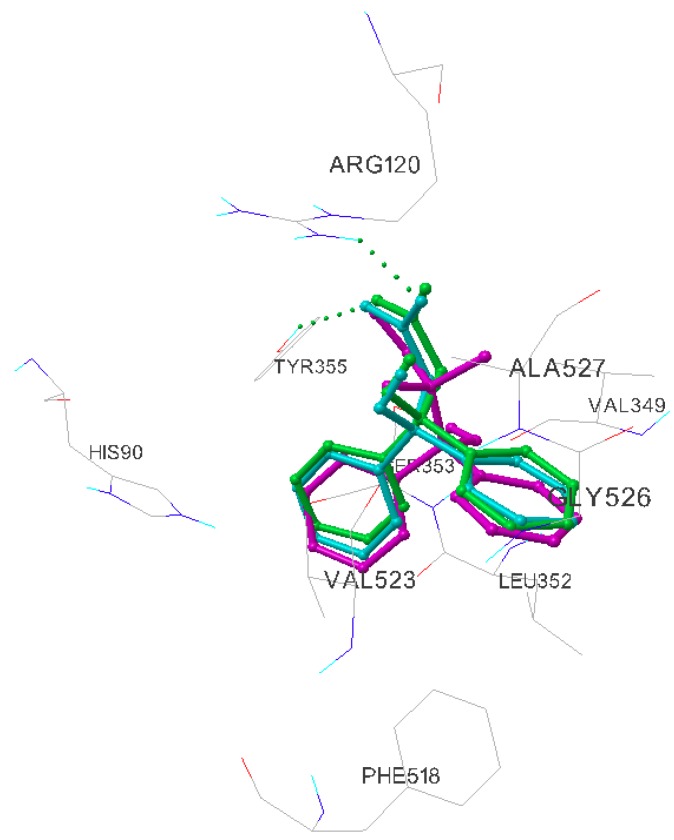
Superimposition of most favourable conformations of compounds **4** (blue), **5** (green) and **6** (magenta) docked into binding site of COX-2 receptor [6].

Acid **6** (at higher doses), like acid **3** (that shows weak dose dependence)**,** possesses the weakest anti-inflammatory activity. It has two methyl groups which send away the carboxylate groups from Arg120 and Tyr 355. Acid **5** possess the strongest anti-inflammatory activity, although it has one methyl group at the α-position. It could be explained by assuming it occupies the position which enables the strongest interaction of carboxylate group with Arg 120 (1.94 Ǻ) and with OH group from acid and N from guanidine group of Arg 120 (3.76 Ǻ), compared to acid **4** (2.06 Ǻ and 5.7 Ǻ) and acid **6** (2.94 Ǻ and 6.66 Ǻ). Also, acid **5** possesses the strongest π→cation interactions with Arg 513 (3.60 Ǻ) compared to acid **4** (3.88 Ǻ) and acid **6** (3.93) (Figure 4).

**Figure 5:**
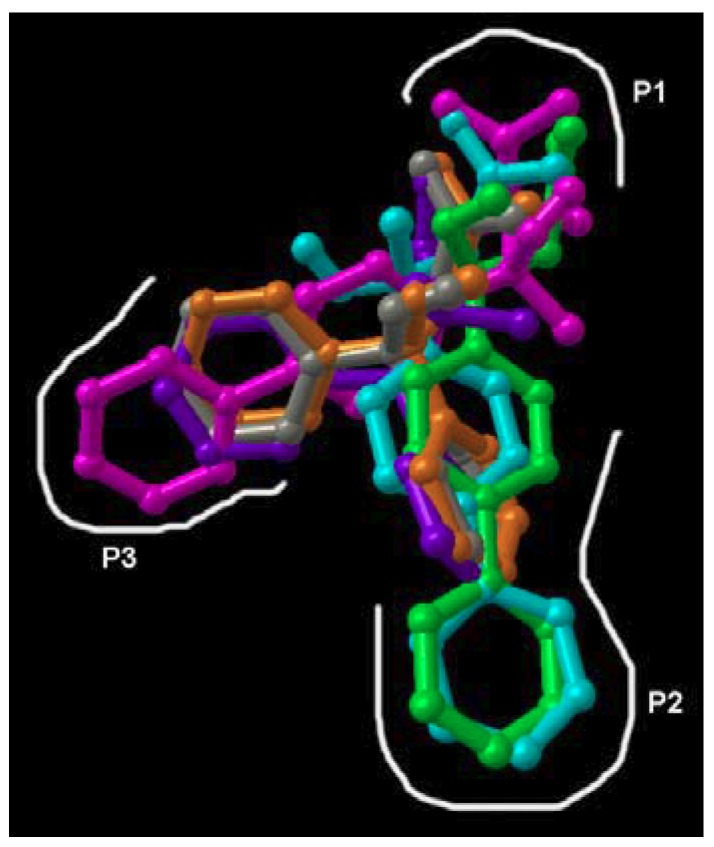
Anchor sites of COX-2 receptors and best docking poses of compounds 1 (green), 2 (blue), 3 (pink), 4 (gray), 5 (orange) and 6 (purple) [6].

The best docking poses are illustrated in Figure 5.

### 2.4. Statistical analysis

Results were expressed as means ± standard deviations (SD). Statistical analysis was done by the Mann–Whitney *U*-test and ANOVA. Differences were accepted as statistically significant when *P <* 0.05.

## 3. Experimental 

### 3.1. Chemistry

In a 100 mL two-necked round bottom flask equipped with CaCl_2_ tube, argon inlet and magnetic stirrer, the α-bromopropanoic acid (0.03 mol), ethyl vinyl ether (3.37 g, 5.00 mL, 0.05 mol) and dry benzene (4–6 mL) were placed and stirred at room temperature for 2 hours. After the evaporation of benzene and excess of ethyl vinyl ether, the residual mixture was distilled under the reduced pressure. In this way 4.5 g (0.02 mol) of 1-ethoxyethyl-2-bromopropanoate was obtained.

In 100 mL three-necked, round-bottom flask, equipped with CaCl_2_ tube, argon inlet and magnetic stirrer, Zn (0.02 mol, 1.30 g), 4-acetylbiphenyl (0.013 mol, 2.54 g), dried THF (40 mL) and small amounts of HgCl_2_ and I_2_ are placed. Previously prepared ester (0.02 mol, 4.5 g) was added from dropping funnel during 30 min under the argon atmosphere. The reaction mixture was cooled in ice bath and constantly stirred magnetically, until whole amount of Zn disappears (3 days). The THF was removed under reduced pressure, followed by addition of benzene (30 mL) and cold 3 M HCl (10 mL). This reaction mixture was cooled at 0 °C in ice bath, and stirred for 3 hours. The organic layers were taken. Obtained aqueous solutions were additionally extracted with benzene. The combined organic extracts were treated with 10% aqueous KHCO_3_ until pH 8 was reached the (hydroxyacid was converted to its potassium salt). The alkaline aqueous solution was extracted by small amount of ether to remove unreacted ketone. This solution was cooled at 0 °C and cold 10% HCl was carefully added to pH 2.5, yielding β-hydroxyacid, as an oil at first, that turned into crystals after prolonged storage at low temperature. The acid obtained in this way was recrystallized from benzene.

### 3.2. Animal studies 

Adult male Wistar rats, weighing 160–180 g, purchased from the Serbian Military Medical Academy Animal House were used in the carrageenan-induced rat paw oedema and the gastric tolerability tests. Experimental groups comprised 6 animals, each. The animals were deprived of food for 18–20 h before the beginning of experiments, with free access to tap water. All experimental procedures and protocols conformed to institutional guidelines for care and use of animals in research No 2/09 (Ethics Committee in research of the Faculty of Pharmacy, Belgrade, Serbia).

### 3.3. Anti-inflammatory activity: carrageenan-induced rat paw oedema test

The carrageenan-induced rat paw oedema test was used as an experimental model for screening the anti-inflammatory activity according to the modified method of Oyanagui and Sato [8]. The tested compounds dissolved in DMSO were administered *p.o.*, throughout the orogastric tube, in doses of 2.5, 5, 10, 15 and 20 mg/kg. ibuprofen (2-[4-(2-methylpropyl)phenyl]propanoic acid), also dissolved in DMSO, was used as a reference, and given in the same dose-range. The control animals were given vehicle DMSO in a dose of 1 mL/kg *p.o.* One hour after the oral administration of the compounds tested, or ibuprofen, carrageenan-saline solution (0.5%) and saline were injected in a volume of 0.1 mL into the plantar surface of the right and left hind paw, respectively. Left paw served as the control one (non-inflamed paw). The animals were sacrificed 3 hours after the carrageenan and saline injection and paws were cut off for weighing. Difference in weight between right and left paw, active drug-treated *versus* vehicle-treated (control) rats, served as an indicator of the anti-inflammatory activity of tested drugs (compounds and ibuprofen). The anti-inflammatory effect was calculated using the equation:

(1) $$\mathrm{Inflammatory}\;\mathrm{response}\;(\%)=\frac{e}{k}\cdot 100$$

where *k* is a difference in the paw weight in the control group, and *e* is a difference in the paw weight in the treated group. On the basis of these results, the corresponding mean effective anti-inflammatory doses (ED_50_) were calculated according to the method of Litchfield and Wilcoxon [9].

### 3.4. Gastric tolerability test

When animals were sacrificed, their stomach were removed and opened along the greater curvature. Lesions were examined under an illuminated magnifier (3×). The intensity of gastric lesions was assessed according to a modified scoring system of Adami *et al.* [10] (0: no lesions; 0.5: slight hyperaemia or ≤5 petechiae; 1: ≤5 erosions ≤5 mm in length; 1.5: ≤5 erosions ≤5 mm in length and many petechiae; 2: 6-10 erosions ≤5 mm in length; 2.5: 1-5 erosions >5 mm in length; 3: 5–10 erosions >5 mm in length; 3.5: >10 erosions >5 mm in length; 4: 1-3 erosions ≤5 mm in length and 0.5–1 mm in width; 4.5: 4–5 erosions ≤5 mm in length and 0.5–1 mm in width; 5: 1–3 erosions >5 mm in length and 0.5–1 mm in width; 6: 4 or 5 grade 5 lesions; 7: ≥6 grade 5 lesions; 8: complete lesion of the mucosa with haemorrhage).

### 3.5. Molecular docking experiments 

To identify potential anti-inflammatory lead among compounds **1–6**, docking calculations were performed using Autodock v4.0.1 [4] into the 3D structure of the catalytic site of COX-2 enzyme (pdb code: 1cx2) and COX-1 enzyme (pdb code: 1eqg).

It should be mentioned that the Lamarckian genetic algorithm implemented in Autodock has been successfully employed to dock inhibitors into the catalytic site of the COX isoenzymes and correlate the obtained binding free energies with inhibitory activities of the compounds. Briefly, we carried out comparative docking experiments of synthesized compounds **1–6** with the known non-selective COX inhibitor ibuprofen. The obtained results were evaluated in terms of binding energy and docking positioning into the catalytic site of COX-2. Docking calculations predicted the binding conformation of SC-558 for COX-2 isoenzyme and binding conformation of ibuprofen for COX-1 isoenzyme with a root main square deviation (RMSD) of 1.44 Δ and 1.48 Δ, respectively, with respect to conformations from X-ray crystallographic studies [6]. 

Structures of all possible stereoisomer forms of ligands were generated using the ChemOffice v7.0 Ultra software package and have been MM2 optimized [11]. Each docking experiment consisted of 10 docking runs with 150 individuals and 500,000 energy evaluations. Other parameters were left to their default values. The search was conducted in a grid of 40 points per dimension and a step size of 0.375 centred on the binding site of enzyme.

## 4. Conclusions

Molecular docking calculations accompanied by *in vivo* biological assay were used to identify potential anti-inflammatory agents among the β-hydroxy-β-arylpropanoic acid class of compounds acting through a COX-2 inhibition mechanism. The obtained results indicate that all compounds possess significant anti-inflammatory activity after oral administration and that compound **5** possesses a strong anti-inflammatory activity, higher than ibuprofen, a standard NSAID. Acids **3** and **6**, with two methyl groups at the α-position possess the weakest anti-inflammatory activity. Tested substances and ibuprofen did not exhibit any significant gastric lesions. Acids prepared from acetophenone have exhibited better anti-inflammatory activity than acids prepared from 4-acetylbiphenyl in the carrageenan-induced rat paw oedema test. It must be stressed-out that compound **4** has anti-inflammatory activity matching that of ibuprofen, and was proven to be non-toxic towards healthy cells [4], and is not chiral, which makes it an interesting synthetic drug.

## Figures and Tables

**Table 1:** Binding energies of top scored solutions as resulting from docking experiments, and corresponding standard biological response of studied compounds.

Compound	Binding energy (kcal/mol)				ED_50_
	COX-1		COX-2		(μM/Kg)
Ibuprofen	R	–6.47	R	–6.21	51.70*
	S	–6.74	S	–6.05	
Compound 1	R	–8.21	R	–8.31	52.71**
	S	–8.16	S	–8.15	
Compound 2	2R3R	–8.61	2R3R	–8.50	70.10*
	2R3S	–8.16	2R3S	–8.49	
	2S3R	–8.15	2S3R	–8.14	
	2S3S	–8.13	2S3S	–8.27	
Compound 3	R	–8.18	R	–8.25	126.82*
	S	–8.13	S	–8.21	
Compound 4		-7.31		–6.92	50.00
Compound 5	R	–7.20	R	–7.33	14.79*
	S	–6.78	S	–7.14	
Compound 6		–7.50		–6.85	74.69

* Result obtained for mixture of enantiomers; **Result obtained for mixture of diastereomers.
